# Faculty members as academic knowledge brokers in Iran's health sector: a social network analysis study

**DOI:** 10.1186/s12961-024-01141-7

**Published:** 2024-04-29

**Authors:** Khadijeh Shabankareh, Ali Hamidi, Mohammad Reza Soleymani, Haniye Sadat Sajadi, Mousa Alavi

**Affiliations:** 1 Health Information Technology Research Center, Isfahan University of Medical Sciences, Isfahan, Iran; 2 Department of Medical Library and Information Sciences, Faculty of Paramedicine, Bushehr University of Medical Sciences, Bushehr, Iran; 3 Knowledge Utilization Research Center, University Research and Development Center, Tehran University of Medical Sciences, Tehran, Iran; 4 Nursing and Midwifery Care Research Center, Isfahan University of Medical Sciences, Isfahan, Iran

**Keywords:** Academic knowledge broker, Evidence-informed policymaking, Knowledge translation, Social network analysis

## Abstract

**Background:**

Interaction between researchers and policymakers is an essential factor to facilitate the evidence-informed policymaking. One of the effective ways to establish this relationship and promote evidence-informed policymaking is to employ people or organizations that can play the role of knowledge brokers. This study aims to analyze the communication network and interactions between researchers and policymakers in Iran's health sector and identify key people serving as academic knowledge brokers.

**Methods:**

This study was a survey research. Using a census approach, we administered a sociometric survey to faculty members in the health field in top ten Iranian medical universities to construct academic-policymaker network using social network analysis method. Network maps were generated using UCINET and NetDraw software. We used Indegree Centrality, Outdegree Centrality, and Betweenness Centrality indicators to determine knowledge brokers in the network.

**Results:**

The drawn network had a total of 188 nodes consisting of 94 university faculty members and 94 policymakers at three national, provincial, and university levels. The network comprised a total of 177 links, with 125 connecting to policymakers and 52 to peers. Of 56 faculty members, we identified four knowledge brokers. Six policymakers were identified as key policymakers in the network, too.

**Conclusions:**

It seems that the flow of knowledge produced by research in the health field in Iran is not accomplished well from the producers of research evidence to the users of knowledge. Therefore, it seems necessary to consider incentive and support mechanisms to strengthen the interaction between researchers and policymakers in Iran's health sector.

## Background

Research in the health field, by knowledge production in terms of providing better technologies and improving people's lifestyle, can lead to social and economic development, in addition to improving the society's health [1]. However, the production of new knowledge will be effective when it is available to stakeholders and used in making decisions and policies. Evidence-informed policymaking (EIPM) helps policymakers use the best available evidence in their policy decisions systematically and transparently. It can lead to more informed decisions, strengthen health systems, and as a result, improve public health outcomes [2].

Activities aimed at using the results of health research in policymaking and practice are known as knowledge translation. knowledge translation experts highlight the importance of both EIPM and practice, and policy and practice-informed evidence generation [3]. However, the gap between knowledge production and its application is considered one of the fundamental challenges of health systems in developing countries, including Iran [4]. These countries face challenges such as the lack of high-quality evidence, limited capacity to evaluate and use evidence, investment policy problems, and structural barriers to evidence use [5]. Research results are not usually available to the policymakers, so, research does not have an objective and noticeable effect on important policies [6]. In such a situation, researchers and policymakers are considered separate communities whose few interactions and different priorities hinder the flow of evidence between them. This gap has been mentioned in various studies, and the interaction of researchers and policymakers has been emphasized as the most essential factor facilitating EIPM [7–9].

One of the effective ways to establish communication between researchers and policymakers is to employ people or organizations as knowledge brokers (KBs) to accelerate the process of knowledge flow by creating an active cooperation network among stakeholders [4]. A KB seeks to transfer knowledge and establish and facilitate communication and interaction between producers and users of knowledge [10]. These people may not be researchers or end users of knowledge. Recently, the notion of academic KB has emerged, highlighting the role of university faculty in facilitating information flow between researchers and policymakers. In addition to producing knowledge, these people can also play a role as mediators of knowledge [11].

In Iran's General Health Policies (GHPs), institutionalization of knowledge production and promotion of evidence-informed decision-making at different levels of policymaking have been emphasized [12]. Therefore, much attention has recently been paid to the concepts of knowledge translation, EIPM, and knowledge brokering in health field. Measures such as the establishment of the National Institute of Health Research, and the Health Policy Council are initiatives that have been presented to promote EIPM in Iran's health system [13]. However, the implementation of EIPM in Iran has faced obstacles, and the use of evidence has not been well institutionalized in the country's health system [14].

One of the most important sectors in modern healthcare systems is the health sector. The World Health Organization has always emphasized the principle that "prevention is better than cure" [15]. Similarly, Iran's health system's motto is “prevention comes before treatment” although it seems that this phrase has not been much considered in practice. In this context, faculty members and researchers at medical sciences universities, specializing in the health field, can take steps towards realizing this motto. These individuals can enhance community health by generating valid scientific evidence and influencing health policies [16]. It requires mutual communication and appropriate interactions between researchers and policymakers in the health field [17].

Individual relationships between researchers and policymakers in the health field for EIPM have been considered in some studies. These studies employed the Social Network Analysis (SNA) method to discern relationships within the scientific network, identifying key individuals or organizations. McAneney et al. drew the stakeholder network structure for academic and non-academic members of the Center of Excellence for Public Health in Northern Ireland using the SNA method. They assessed the quality and extent of these relationships, exploring their impact on research influencing policy and practice. Additionally, they delved into researchers' perspectives on knowledge brokering and knowledge translation [18]. Yousefi-Nooraei et al. depicted the information search network concerning the integration of research evidence into practice among staff in the Canadian public health department. They investigated the nature of connections between staff, network structure, key actors, and KBs and their characteristics [19]. The role of academic KBs was first discussed in Jessani's doctoral dissertation [11]. The author drew a communication network for the faculty members of public health schools and policymakers in Kenya and defined KBs and their characteristics [11]. In a separate study, Jessani et al. investigated the connections between faculty members at the Johns Hopkins Bloomberg School of Public Health and policymakers at the city, state, federal, and global levels [20].

Studies conducted in the field of EIPM in Iran have often evaluated the impact of health research [21], obstacles and challenges of EIPM [22], and strategies to strengthen EIPM [13, 14, 17, 23]. All these studies have emphasized the lack of communication and mutual trust between researchers and policymakers and the need to strengthen the interaction between these two groups. To our knowledge, there is no specific research addressing the relationship between researchers and policymakers in Iran's health sector, with a particular emphasis on the role of faculty members as KBs. Therefore, the current study has sought to depict the current situation by analyzing the communication network and interactions between researchers and policymakers in Iran's health field and identify key people who can play a role as academic KBs.

## Methods

### Design and setting

This study was a survey, which is part of a wider research with the aim of explaining the role of academic KBs in Iran's health sector. We used the SNA method to draw the communication network between faculty members and policymakers in the health field in Iran [24]. SNA is a quantitative method for analyzing data in social networks that studies the relationships between actors, including individuals or organizations, and how they communicate to access intellectual, financial, social, and other resources [25]. Additionally, this method serves to identify influential individuals, organizations, and KBs within the network [20, 26]. SNA reveals the fine interpersonal and interdepartmental communication structure, which cannot be visualized using conventional surveys [19].

### Participants and sampling

The research population involved the faculty members specializing in the health field in top ten medical universities in Iran, under the MOHME, including Tehran UMS, Shahid Beheshti UMS, Mashahd UMS, Tabriz UMS, Iran UMS, Shiraz UMS, Isfahan UMS, Ahvaz Jundishapur UMS, Mazandaran UMS, and Kerman UMS. The faculty members' names were extracted from the Iranian Scientometrics Information Database.^1^ Accordingly, 302 people with degrees in health education and promotion, public health, occupational health, environmental health engineering, and epidemiology who had at least one article in the Scopus, regardless of the faculty or research center where they worked, were included in the study. Therefore, the sampling method was non-probability, purposeful and total population sampling.

### Data collection

For data collection, a sociometric questionnaire, commonly employed in studies focused on social relations, was utilized [27]. Data collection was done January to August 2020 by visiting in person or sending an online questionnaire via email. In order to increase the response rate to online questionnaires, reminder e-mails were sent to people several times at ten-day intervals. In addition to providing demographic information, this questionnaire provided the necessary data to draw the communication network. In the questionnaire, the respondents were asked to name the people with whom they interacted in order to apply research evidence in health policies. According to previous studies, in the sociometric survey, the maximum number of people introduced by the respondents in a network varied between 5 and 7 people [19, 28]. Therefore, in the present study, the respondents were asked to introduce a maximum of 7 people in each of the three categories determined in the questionnaire. The three categories of relationships between researchers and policymakers were: (1) direct relationship with policymakers, (2) relationship with policymakers through peers, and (3) researchers as intermediaries between policymakers and peers.

### Data analysis

UCINET, version 6.716, and NetDraw 2.173 software were used for network visualization. To achieve this, initially, data from the sociometric questionnaire were input into Excel. Then, the reported connections between each respondent and their alters were recorded in a relational matrix in UCINET. Subsequently, the data file was saved and analyzed. For simplicity and to maintain the confidentiality of the respondents, the names of people were replaced with unique IDs in the matrix. Each row or column in the matrix represents one of the nodes (i.e., the network members). In the matrix, the numerical value of the relationship between nodes was entered. In this way, if there is a connection between two nodes, the number 1 is entered, and if there is no connection, the number 0 is entered. In order to reduce the possibility of data loss, those groups of the research population who did not complete the questionnaire, provided that another member of the network introduced them as peers, were also included in the network.

After drawing the network, KBs were selected based on the following three factors:

1. Number of links with policymakers;
2. Number of links with academic peers;
3. Individuals' positions in the network, serving as links between other nodes, were assessed using the Betweenness Centrality index in the UCINET. In other words, in this research, Indegree Centrality, Outdegree Centrality, and Betweenness Centrality indicators were used to determine key actors or KBs in the network.

A high Indegree index indicates the person's reputation, which shows that many people pay attention and refer to this node. A high Outdegree index suggests authority, signifying that nodes of this kind can disseminate information more rapidly [29]. The Betweenness Centrality index of a node expresses the importance of that node in the flow of information passing from one part to other parts of the network. Thus, it can show the ability of a node to create connections between the other nodes [30]. In order to determine KBs, people were first ranked based on the indicators of Indegree Centrality (number of connections with peers), Outdegree Centrality (number of connections with policymakers and number of connections with peers), and Betweenness Centrality, and then the scores were normalized. Due to the limited number of qualified people, people who were in the top 50% in all four indicators were selected as KBs. The reason for choosing these indices was that the academic KBs did not gain this position only as a channel to control the flow of information (which is determined using the Betweenness Centrality index). However, their position could also be due to their reputation and popularity among their peers and their effect on policymakers through direct or indirect communication [28].

In order to determine the key policymakers in the network, the frequency of repeating the names of the policymakers introduced by the faculty members, according to the information from the sociometric questionnaire, was considered. Since this frequency varied from 1 to 5, the Median number was considered an indicator for determining key policymakers. As a result, policymakers mentioned three times or more were chosen as key policymakers in the network. These policymakers had the highest interaction with faculty members, as reported by respondents. After drawing the network, the structural characteristics of the network, including prevalence, depth, and effective density, were also calculated.

The prevalence of connections between network members has been calculated in two ways: (1) absolute prevalence, which includes the number of faculty members participating in the research who have had contact with at least one policymaker, and (2) Relative prevalence, which is the ratio of faculty members who have had contact with at least one policymaker to all faculty members participating in the research [20].

The depth of communication indicates the degree of overlapping of communication between faculty members and policymakers, and peers. In other words, it is the ratio of joint members with more than one connection with academic faculty members of the research population to all policymakers and peers introduced in the network [28].

Network density is defined as the ratio of the number of all existing links to all possible links [31]. This index, which represents the degree of network correlation, can provide insight into the speed of information dissemination among nodes and the amount of social capital or social restrictions of actors [32]. Usually, the higher the density of the network, the more direct relationships there are between members, [29] and the more likely knowledge is to flow in the network [33]. Because, in the present study, the respondents were limited to introducing seven people in each of the three categories of communication, the concept of effective density has been used.

## Results

One hundred fifty-two people (50.33%) of the research population answered the sociometric questionnaire, whose demographic information is presented in Table 1.

**Table 1:** Respondents overview

Respondents (% of eligible)	Gender		Academic Position				University major				
	M	F	Instructor	Asst. Prof	Asst. Prof	Prof	Public health	Environmental Health Engineering	Occupational Health Engineering	Health Education and Promotion	Epidemiology
152 (50.33%)	106 (69.74%)	46 (30.26%)	6 (3.95%)	73 (47.03%)	37 (24.34%)	36 (23.68%)	2 (1.32%)	49 (32.24%)	36 (23.68%)	36 (23.68%)	29 (19.08%)

### Individual network

**Fig. 1:**
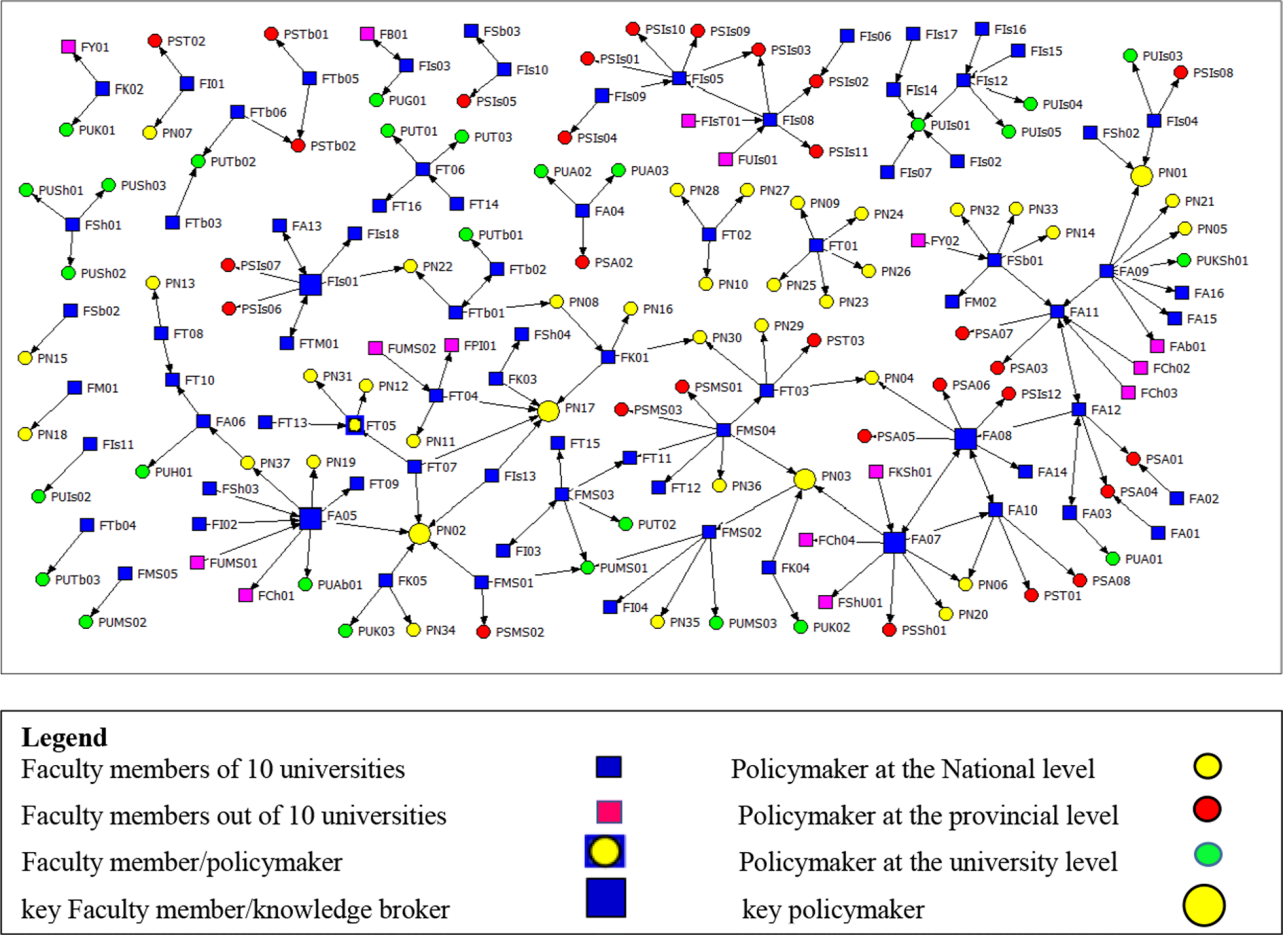
Academic-policymaker network for top ten Iranian universities of medical sciences

Fifty-six (36.84%) of the respondents, asserted their interaction with at least one policymaker. This interaction aimed to share the results of their research. Thirty-six (23.68%) of the respondents, indicated having experience as decision-makers. The communication network of faculty members and policymakers in Iran's health sector is shown in Fig. 1. This network has 188 nodes consisting of 94 university faculty members and 94 policymakers at three national, provincial, and university levels. One of the faculty members with the code FT05, who completed the sociometric questionnaire as a researcher, also has a policymaking role at the national level. Due to the fact that he was introduced as a policymaker by some members of the research population, he is considered as a policymaker here.

In the drawn network, faculty members are represented as squares, and policymakers as circles. The connection between the members of the network is also shown with directional lines, so that the connection between faculty members and policymakers is shown as one-way lines (from faculty members to policymakers) and the connection between faculty members and peers is shown as one-way or two-way lines (Fig. 1).

**Fig. 2:**
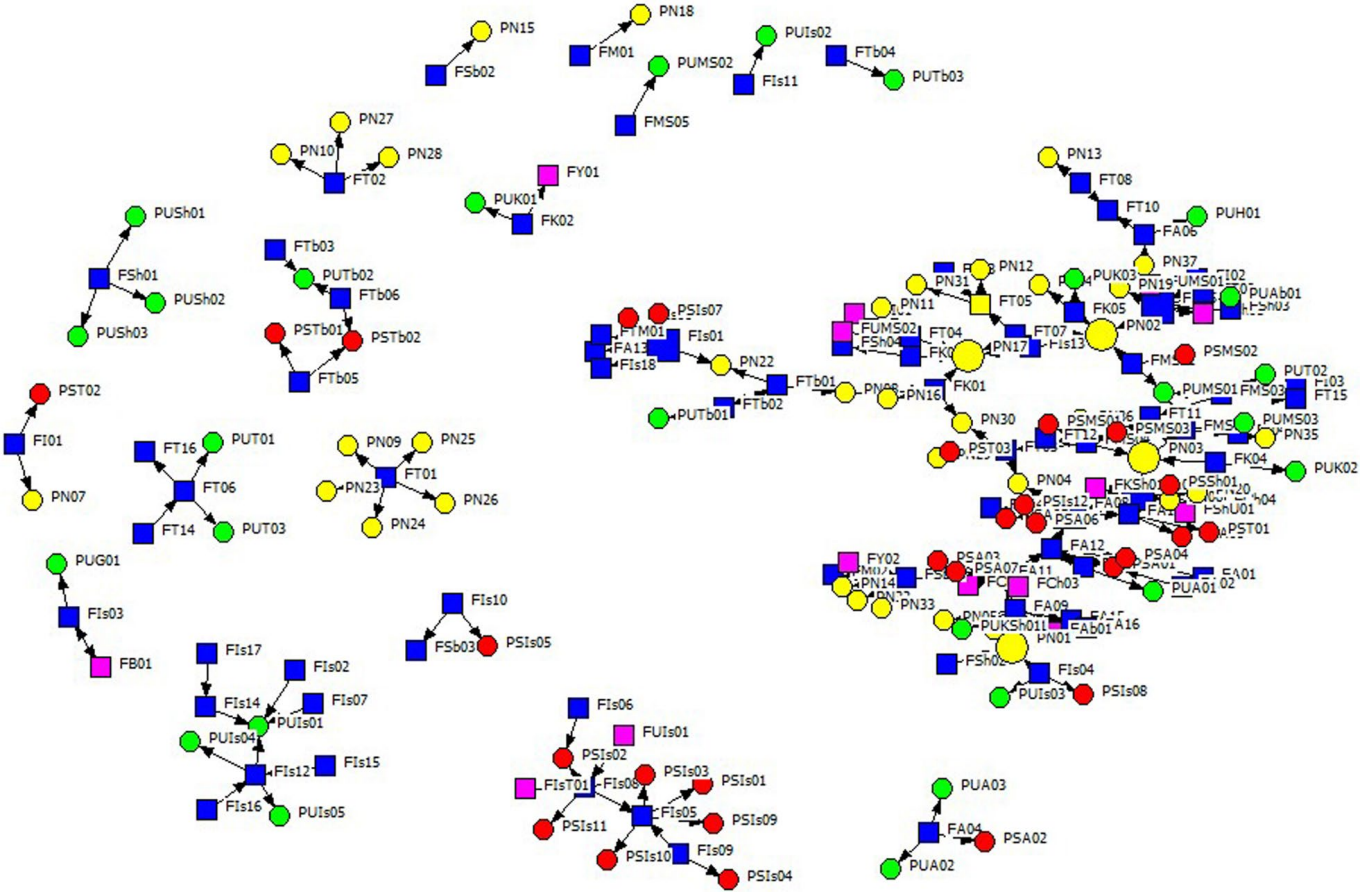
Subgraphs of academic-policymaker network

In the drawn network, 114 nodes, connected through 119 links, formed the main component. A component is defined as the largest connected subgraph. Other nodes were outside the main component and separated into small subgraphs. The network drawn based on the existing subgraphs is shown in Fig. 2.

In the network, there were a total of 177 links. Specifically, 125 links were established with policymakers. Moreover, 52 links were established with peers. Of these, nine links were two-way links. It should be noted that all policymakers and peers (regardless of employment status, field of study, and university of employment) who were introduced by the faculty members were retained in the network for complete analysis. However, 96 respondents had no connection with the policymakers or their peers to share the results of their research with the policymakers, were considered isolated nodes and were not displayed in the network.

### Organizational network

**Fig. 3:**
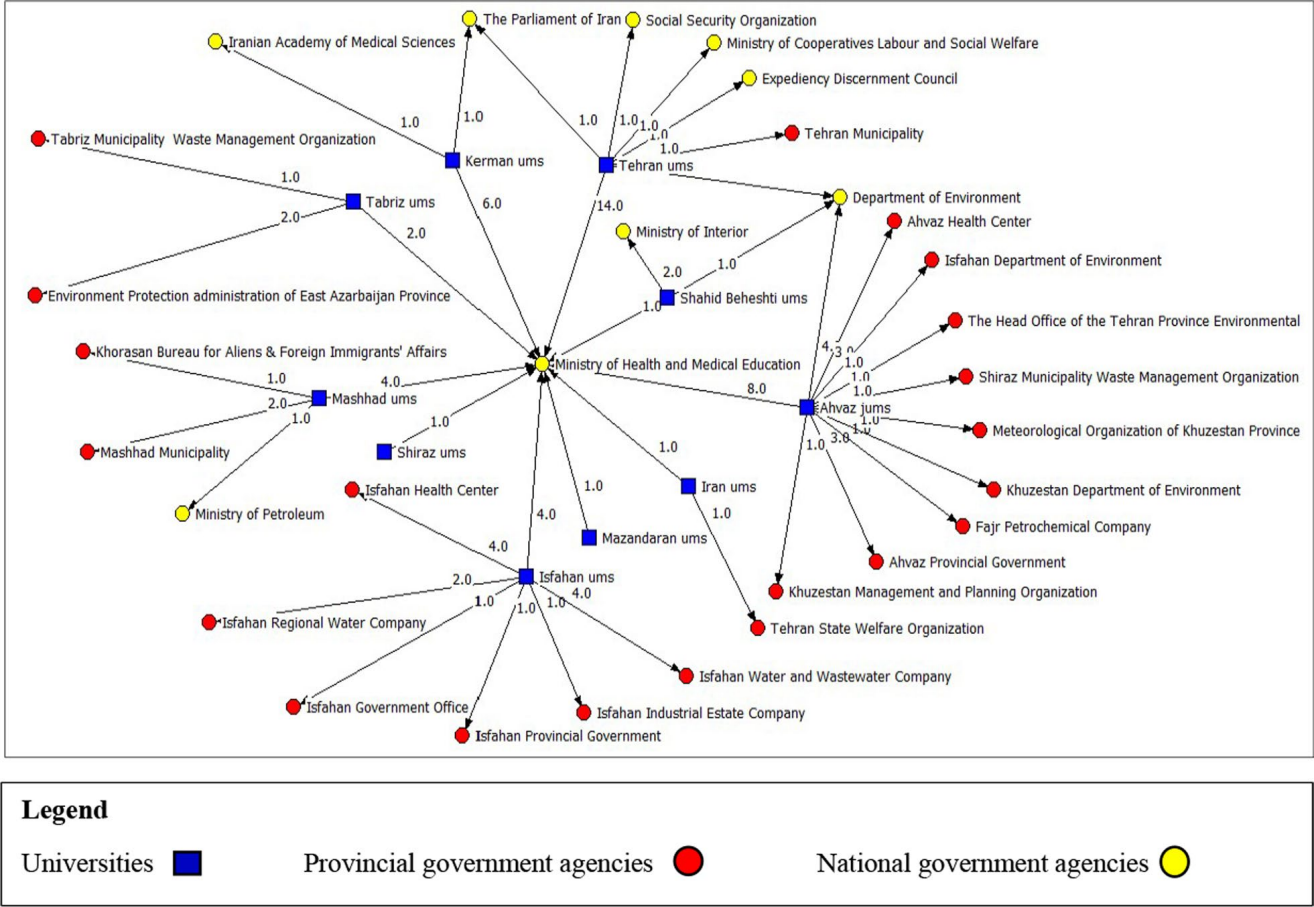
Organizational network at national and provincial level

Faculty members were linked with policymakers from nine organizations at the national level. Furthermore, connections were established with policymakers from 21 organizations at the provincial level. Thirty-three relationships were also established with policymakers at the level of medical sciences universities, including presidents and vice-chancellors of universities. The highest number of connections at the national level was with policymakers from the MOHME (42 connections), so faculty members in all ten surveyed universities had connections with policymakers from this organization. Most of the connections at the provincial level were established with provincial health centers (7 connections) and the Department of Environment (5 connections). Respondents from all universities in the survey were linked with at least one organization at the national level. Notably, Tehran University of Medical Sciences held the largest share among these connections, establishing links with six organizations. However, at the provincial level, the faculty members of 4 universities had no connection with the policymakers. Regarding the variety of organizational connections at the provincial level, Ahvaz Jundishapur University of Medical Sciences had the best situation. It was the only university whose faculty members had connections with policymakers from other provinces (Fig. 3).

### Network characteristics

**Fig. 4:**
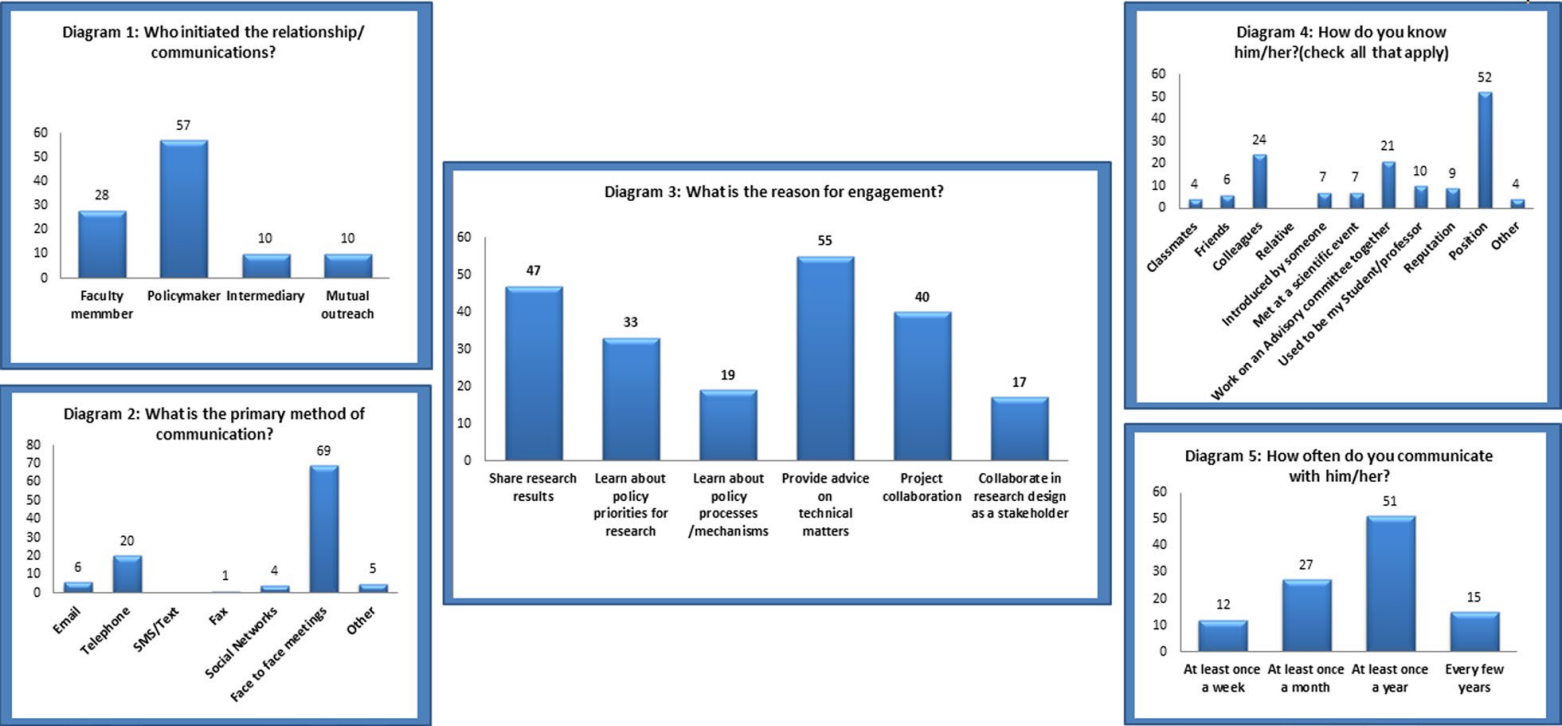
Interactive features of academic-policymaker network

The structural characteristics of the network, and their associated formulas are given in Table 2. Information on interactive features is also provided in Fig. 4.

**Table 2:** Measures of network characteristics

Metric	Result	Formula
Network size	188	Network size = [total number of nodes in a network] The nodes represent all the respondents and decision makers/ their peers in each network (excluding isolates)
Inclusiveness	0.66	Inclusiveness = [network size/ (network size + isolates)] *Isolates are respondents who indicated no alters and therefore do not appear in the network as ‘connected’
Average degree	0.94	Average degree = [total no. of ties in network]/[network size]
Effective density	0.15	Effective density = [total no. of ties in network]/[ (total no. of respondents in network * maximum number of nominations requested)] *Maximum numbers of nominations was seven at each level
Prevalence of relation		
Absolute prevalence	56	Absolute prevalence = [total no. of respondents with ≥ 1 alter)]
Proportionate prevalence	0.37	Proportionate prevalence = [total no. of respondents with ≥ 1 alter)/ total no. of respondents]
Breadth		
Contacts	132	Breadth of contacts = total no. unique alters (nodes) in a network
Relations	177	Breadth of relations = total no. relationships (ties) in a network
Average	1.16	Average breadth of relations = average number of ties per respondent = [total no. of ties in network/ total no. of respondents]
Depth	0.34	Depth = [ (total number of ties – total number of alters)/ total number of alters]

Jessani et al. [20]

The findings of the investigation of the structural characteristics of the network indicated the limited direct relationships between the members and the low correlation of the network, so the effective density of the network was about 0.15. In other words, 56 respondents, considering the limit of 7 connections in each category (connections with policymakers and peers), were potentially able to establish 1176 connections. However, the total number of links in the network was 177 links (Table 2).

The findings related to the network interactive features showed that the request for interaction was often made by policy makers (54.29%). The primary communication between researchers and policy makers has been done mostly in face-to-face meetings (65.72% (. The interaction was mostly aimed to provide advice to policymakers about technical issues (26.07%), sharing research results (22.28%), and requesting research or collaboration in a project (18.96%. ( The researchers acquaintance with policymakers was mostly due to the organizational position of the policymaker (49.52%), being a colleague (22.86%), and working in an advisory committee (20%. ( In 48.57% of cases, researchers had contact with policy makers at least once a year (Fig. 4).

### Key actors in the network

#### Academic KBs

Among the 56 faculty members who had relationships with policymakers or peers, 24 individuals met the criteria established in the research and were eligible to be selected as KBs, Subsequently, four individuals were chosen as academic KBs, as outlined in Table 3. One was female. Regarding academic position, three were professors, and one was an associate professor. Two had a PhD degree in environmental health engineering, one had a PhD degree in public health, and one had a PhD degree in health education and promotion. They had been at their respective organization between 6 and 28 years. All four KBs had acknowledged that they have been or are currently playing a role as policymakers in the health field at the national or provincial level.

**Table 3:** Centrality measures across the four SNA-identified academic knowledge brokers

Identification Code	Outdegree to policymaker	Outdegree to policymaker normalized*	Indegree from peers	Indegree from peers normalized**	Outdegree to peers	Outdegree to peers normalized*	Peer and PM betweenness centrality	Peer and PM betweenness centrality*** (normalized)*100	Total Score
FA08	4	57.14	3	42.85	3	42.85	186	0.529*100 = 52.9	195.74
FA07	4	57.14	2	28.57	4	57.14	125	0.354*100 = 35.4	178.25
FA05	4	57.14	3	42.85	2	28.57	27	0.075*100 = 7.5	136.06
FIs01	3	42.85	2	28.57	3	42.85	10	0.028*100 = 2.86	117.13

*Outdegree to policymaker/peers is normalized to 7 potential nominees: #alters/7

**Indegree from peers is normalized to 7 potential nominees: #alters/7

***Peer&PM betweenness centrality is normalized by UCINET

### Key policymakers

A total of 94 policymakers at three national, provincial, and university levels were nominated by the respondents. Specifically, the names of 75 policymakers were mentioned once. Furthermore, the names of 19 policymakers were cited multiple times. This included 11 at the national level, five at the provincial level, and three at the university level. The highest frequency of repeating the name of a policymaker in the network was five times, which was related to two policymakers at the national level. Based on the criteria considered, four policymakers at the national level and two policymakers at the university level were selected as key policymakers (Table 4).

**Table 4:** SNA-identified key policymakers

Identification code	Indegree from Faculty members	Organization
PN17	5	MOHME
PN02	5	MOHME
PUIs01	4	Isfahan University of Medical Sciences
PUMS01	3	Mashhad University of Medical Sciences
PN01	3	MOHME
PN03	3	MOHME

## Discussion

In this study, using the SNA method, the current state of personal communication between researchers and policymakers in the health field in Iran was depicted. The results show that there are several faculty members in communication network who can play a role as academic KBs in Iran's health sector.

The findings showed a low interaction between researchers and policymakers in the health field in Iran. The low effective density of the network also confirmed that there are not many direct relationships between the network members. So, the possibility of the flow of knowledge obtained from research in the network is low. It indicates that the researchers have little influence on decision-making process in Iran's health sector. Yousefi-Nooraei et al. also showed the existence of a local communication network with low density and limited connections between public health workers in Canada [19]. However, based on Jessani et al., the frequency of relationships and the degree of influence of public health schools of the Johns Hopkins University in the United States on decisions were evaluated as appropriate [20]. In another study, Jessani et al. also reported that the level of interaction between faculty members of public health schools and policymakers in Kenya is favorable [28].

The factors such as lack of mutual trust between researchers and policymakers, lack of attention to long-term research programs and need-based research, and the existence of inappropriate criteria for evaluating the performance of policymakers and academic members in Iran can be considered as reasons for their lack of interest in engaging in EIPM [22]. In addition, the lack of appropriate incentives for researchers to participate in activities related to the knowledge translation and application of research results can cause researchers to be reluctant. As a result, they are often content only with the production and dissemination of knowledge in the form of articles. McAneney et al. also showed that academic members were less concerned with knowledge transfer as a personal goal than non-academic members of the UKCRC Center of Excellence for Public Health in Northern Ireland. They mainly expected this center to generate new knowledge and had fewer expectations about health interventions and influence on health policies [18]. Changing the incentive structure based on the publication of research in journals with a high impact factor towards publication in specialized media that have a greater chance of influencing policy and practice can make academic researchers more willing to engage in activities related to EIPM [34].

Examining the degree of overlapping of communication between faculty members and policymakers, and peers, which is referred to as the depth of communication, indicated the low depth of individual and organizational communication in the network. It shows that most of the communication in the network relies on interpersonal relationships, and probably, there is no clear organizational structure to communicate between health professionals and policymakers. Jessani et al. showed that despite the breadth of connections amang the faculty members at the Johns Hopkins Bloomberg School of Public Health in the United States, the depth of the relationships was generally low, which, together with the heterogeneity of the communication between the faculty members/policymakers, shows that most of the communication relies on interpersonal connections. The diversity of relationships and the creation of stable institutional structures for interaction with policymakers can increase the flexibility of networks at the organizational level and overcome the challenge of dependence on individuals. At the same time, the failure to achieve this can cause the network to disintegrate [20].

The organizational network analysis also demonstrated the low level of organizational communication between universities of medical sciences and decision-making organizations in Iran's health sector. According to Doshmangir et al., to ensure the successful implementation of EIPM, knowledge-generating organizations should pay special attention to knowledge management and organizational communication management, and increase their communication with their audience, including decision-makers and policymakers. In this regard, removing institutional barriers and using mechanisms and networks for effective interaction between producers and users of knowledge at macro and intermediate (organizational) levels will be effective for individual and institutional capacity building [23]. Accordingly, creating opportunities for formal and informal interaction, encouraging researchers and policymakers in organizations, and giving organizational support to faculty members willing to interact with policymakers can increase the role of universities of medical sciences and, consequently health research in EIPM. Since, based on the results, the initial communication between researchers and policymakers has mostly been done in face-to-face meetings; it is recommended to hold regular meetings to strengthen the interaction and exchange of information between researchers and policymakers in the health field, by the MOHME as well as other policymaking authorities at the national and provincial levels, and the universities and research centers.

In addition to the need for organizational support, prior acquaintance between researchers and policymakers seems to be an essential factor in forming communication and interaction in the direction of EIPM. The results of the research showed that prior acquaintance between the faculty members and the policymakers was mainly due to personal familiarity, and more than half of the connections were expressed and channeled through being a colleague, having a history of working together in an advisory committee, professor-student relationship, friendship and classmate relationship. As Kotim et al. also showed, actors are more likely to cooperate in the network when they have already cooperated [35]. Jessani et al. also showed that policymakers prefer to get the experts' opinions through personal and trusted contacts. However, they acknowledge that a delicate balance between using individual relationships and creating more stable organizational partnerships is required [36]. The results showed that a low rate of these communications was made by a third party who could potentially play the role of a KB. It indicates the existence of a gap in the role of knowledge brokerage in Iran's health sector. According to McAneney et al., academic members attached less importance to the role of KBs and interdisciplinary collaboration [18].

Additionally, it seems that researchers with executive backgrounds or current executive positions as decision-makers can interact more with policymakers in the direction of EIPM. All four KBs identified in this research admitted that they have played or are playing a role as policymakers in the health field at the national or provincial level in the past or at present. On the other hand, two of the policymakers who had the most connections with the researchers in the network were themselves faculty members of the health educational departments of the studied universities, which can be a justification for their more excellent communication with the faculty members of the research population. Therefore, having the dual role of researcher/policymaker can help to facilitate EIPM and interaction.

A noteworthy point in the drawn network was the presence of some academic faculty members in the network who had a relatively large number of direct connections with policymakers, especially at the national level, but had no or few connections with their peers in order to share research results with policymakers. For example, node FT01 had the most relationship with policymakers. However, it did not play a role in facilitating communication between peers and policymakers. So, it lacked the conditions of knowledge brokering in this research. Jessani et al. showed that although people with strong relationships with policymakers are less likely to contact their peers to access policymakers, they are more likely to receive more requests from their peers to help and connect them with policymakers [28]. Since these people can provide potential opportunities to involve research in policymaking through connection with policymakers, by identifying these people and training them to acquire the necessary skills, their influence can be used at the service of knowledge brokering to facilitate communication between researchers and policymakers.

### Research limitations

Because, about half of the members of the research population did not answer the sociometric questionnaire and some of the respondents refused to mention the names of policymakers and their peers (and therefore were excluded from the study), the drawn network cannot give a complete picture of the existing relationships between faculty members and policymakers in Iran's health sector. Besides, since the sociometric questionnaire was completed only by the faculty members of the research community and no survey of policymakers and peers was conducted, we cannot confirm the bidirectionality of relationships.

## Conclusions

Based on the results, it seems that the flow of knowledge produced by research in the health field in Iran is not realized well from the producers of research evidence to the users of knowledge. Accordingly, it seems necessary to consider incentives and support mechanisms to strengthen the interaction between researchers and policymakers in Iran's health sector. In this context, measures such as creating and strengthening individual and organizational incentives to produce valid and effective scientific evidence, increasing research cooperation between researchers and policymakers, revising the regulations for the scientific promotion of the academic community, providing educational programs to promote the culture of using research evidence in policymaking, and providing formal and informal interactions between researchers and policymakers to encourage their participation in activities related to EIPM are recommended. Formulating strategies to institutionalize the culture of knowledge translation and EIPM in the strategic planning of universities and research institutes can help structure this process.

Moreover, by examining the communication network of faculty members in different universities, it is possible to identify people who can play the role of KB. By organizing these people in the form of knowledge brokerage groups, their capabilities can be used to facilitate communication between researchers and policymakers and to apply the results of research in the health field. To achieve this goal, the MOHME and Iranian medical sciences universities should establish formal and structured knowledge brokering activities. One way to accomplish this is by establishing a knowledge translation committees. To discover and benefit from existing relationships between researchers and policymakers, SNA studies should be conducted regularly by universities. This will help identify and utilize the individual and organizational capacities needed to realize this goal. Identifying the existing gaps in the communication network helps in the necessary planning to solve these gaps and to create and develop targeted networks. It is suggested to study the communication network status of researchers and policymakers in other fields related to Iran's health system.

## Data Availability

Not applicable.

## Abbreviations

EIPM: Evidence-informed policymaking
KBs: Knowledge Brokers
MOHME: Ministry of Health and Medical Education
SNA: Social Network Analysis
UMS: University of Medical Sciences

## Footnotes

1 https://isid.research.ac.ir [Accessed Jan 2020].
